# MusA: Using Indoor Positioning and Navigation to Enhance Cultural Experiences in a Museum

**DOI:** 10.3390/s131217445

**Published:** 2013-12-17

**Authors:** Irene Rubino, Jetmir Xhembulla, Andrea Martina, Andrea Bottino, Giovanni Malnati

**Affiliations:** Department of Control and Computer Engineering, Polytechnic of Torino, C.so Duca degli Abruzzi 24, 10129 Torino, Italy; E-Mails: irene.rubino@polito.it (I.R.); jetmir.xhembulla@polito.it (J.X.); andrea.martina@polito.it (A.M.); giovanni.malnati@polito.it (G.M.)

**Keywords:** marker recognition, indoor positioning, navigation system, indoor LBS, mobile guides, multimedia, context-awareness

## Abstract

In recent years there has been a growing interest in the use of multimedia mobile guides in museum environments. Mobile devices have the capabilities to detect the user context and to provide pieces of information suitable to help visitors discover and follow the logical and emotional connections that develop during the visit. In this scenario, location based services (LBS) currently represent an asset, and the choice of the technology to determine users' position, combined with the definition of methods that can effectively convey information, become key issues in the design process. In this work, we present Museum Assistant (MusA), a general framework for the development of multimedia interactive guides for mobile devices. Its main feature is a vision-based indoor positioning system that allows the provision of several LBS, from way-finding to the contextualized communication of cultural contents, aimed at providing a meaningful exploration of exhibits according to visitors' personal interest and curiosity. Starting from the thorough description of the system architecture, the article presents the implementation of two mobile guides, developed to respectively address adults and children, and discusses the evaluation of the user experience and the visitors' appreciation of these applications.

## 1. Introduction

Multimedia interactive guides are rapidly replacing the old-fashioned audio and textual guides in city and museum tours. Nowadays, the availability of low-cost and powerful consumer mobile devices, providing rich interaction with a large variety of multimedia contents, and the availability of fast wireless communication networks are boosting the appearance of mobile applications dedicated to supporting tourist mobility [1–3]. The increasing potential provided by mobile guides has been underlined by several surveys [4–7]. Nowadays, their functionalities cover a wide range of services aiming at helping users to create meaningful connections with both physical and digital environments.

When developing an application to be deployed in a museum context, a set of preliminary questions involving both the curators and the developers needs to be answered: what is the objective of the application? How can information be effectively communicated to the target users? How to cope with users' different cultural background, interests and age? When the mobile indoor dimension of such an application is taken into account, additional issues arise, such as how to deliver contextual information to visitors related to the artworks or locations in their surroundings and how to help them follow the physical path of the exhibit and find their way through complex museum halls that they are probably visiting for the first time

In order to answer these and other research questions, in this paper we present Museum Assistant (MusA), a general framework offering a set of predefined tools and functionalities that can be used to develop flexible and compelling multimedia interactive guides for mobile devices. These guides are aimed at helping to provide a meaningful exploration of exhibits and cultural venues according to the personal interest and curiosity of visitors, supporting them with contextualized information while, at the same time, providing personalized ways of managing their visit path and access to cultural contents.

The key feature of MusA is the capability to accurately identify the position of the visitor within the museum. Based on this information, location based services (LBS) can be provided. The main services offered are indoor positioning and navigation. In a museum it is easy to get lost, especially if its floor plan is complex, and the relevant literature has frequently underlined that orientation and way-finding represent an issue for both museum curators and visitors [8]. Considering that patterns of circulation and attention ultimately influence visitors' cultural and leisure experience [9], providing solutions that help visitors orient themselves both physically and cognitively seems particularly important for cultural institutions that aim at fulfilling their educational mission.

Another relevant feature of MusA is the possibility to deliver visitors additional rich multimedia contents that can provide insights about their surrounding environment and the works of art they are seeing through textual commentaries, high-resolution images, videos and audio contributions that can be accessed and browsed through the device display. Different thematic exhibits can be designed by the curators and managed by MusA. Thus, users are provided with the possibility to choose at any time their visit path according to their interests and curiosity. A thematic path can help to reduce barriers between the museum and the visitors, otherwise overwhelmed by the quantity and variety of contents that are hard to process in a short visit time, improving the learning process and the recall of information from the exhibit. Furthermore, such a structure allows one to include a cultural object in different thematic paths, and thus, to communicate its information in different ways and styles, according to the specific perspective of the narrative designed by the curators or to the specific audiences targeted by the themes. As a further option, visitors can create their own tour, by selecting the works of interest to them and then being guided to selected destinations. Finally, the knowledge of the user's position inside the building allows one to exploit location-based games in the educational process.

The structure of MusA allows the rapid prototyping and development of mobile guides for museums and cultural sites. As an example, besides describing MusA functionalities, we present the implementation of two mobile applications based on this framework and focused on the Palazzo Madama-Museo Civico d'Arte Antica, an ancient art museum and UNESCO-listed historic residence located in the city center of Turin (Italy). The first application, called “*Step by step*”, is a mobile guide that offers different thematic paths for visiting the museum collections, while the second, “*Intrigue at the museum*”, is a location-based game, specifically addressed to children aged 7–13, which aims at making the museum visit more appealing, interesting and fun for them. Both the developed applications were thoroughly evaluated in order to investigate the overall appreciation of their functionalities, usability and effective capability to support an edutainment process in a museum context.

The rest of the paper is organized as follows: In Section 1.1 we offer an overview of the related works. Section 2 presents the overall architecture and characteristics of MusA. In Section 3 we describe the two prototypical applications based on the MusA framework. Finally, results of the user tests are presented and discussed in Section 4, while Section 5 draws the conclusions and sketches future works.

### 1.1. Mobile Museum Guides: Related Works

Indoor positioning and navigation on mobile phones have become very popular in recent years, due to the increasing demand for indoor LBS and to the widespread diffusion of low cost consumer devices capable of supporting them. Several applications have been already presented and many others are foreseen. To name a few, indoor navigation can help users find their way through complex buildings, like large malls, hospitals, airports or tradeshows; additionally, conference attendees can receive information to organize their timetable [10] and students at universities can navigate from classroom to classroom according to their lecture schedules [11].

In a museum, the knowledge of the visitors' location allows curators to enhance their cultural experience, by delivering them contextualized multimedia content related to the observed artworks or to the surrounding location [12]. In this framework, the main problem to solve is getting accurate users' location and orientation data. The unavailability inside buildings of GPS signals, or their unreliability, results in the need to deploy a suitable technology for indoor positioning. Several approaches presented (surveyed in [13]) are based on the coverage of the area with various sensor types, exploiting: (i) different radio signals, such as Wi-Fi [14,15], RFID [16–21] and NFC [22]; (ii) perturbations of the Earth's magnetic fields [23]; (iii) modulated infrared lights [24] or (iv) ultrasounds [25].

Though providing a set of valuable solutions, many of these techniques require planners to face some drawbacks. First, they often entail installing and maintaining complex and often expensive physical and electronic infrastructures to guarantee the pervasive availability of the sensed signals. This aspect represents an issue in historic buildings such as museums, where the provision and deployment of these infrastructures can be difficult due to conservation and financial or structural reasons. Second, these positioning techniques might rely on special purpose devices to sense the localization signals, thus ruling out a significant part of off-the-shelf mobile devices. Third, indoor LBS requires a very high precision, since errors not exceeding few meters are necessary to tell one corridor from the other and one floor from the next. This is a matter of choosing the type of sensing signals, as some could be inherently inaccurate. Finally, navigation planning requires not only a precise localization but also a correct orientation, which still turns out to be a big unsolved issue depending on the type of sensing signals used by the system.

Vision-based localization system can address in efficient and satisfactory ways most of the previous points. They rely on the extraction of image features, like points, edges or regions, which are matched with those in a reference database to compute device position and orientation. In order to make the feature detection and matching problems easier, most of the approaches rely on visual markers, depicting specifically designed two-dimensional patterns, such as QR codes, bar codes or tags similar to those used in Augmented Reality applications. Alphanumeric information can be encoded into the marker and used as key to obtain the fiducial position and orientation from a database. Finally, the peculiar visual appearance of the markers makes them easily identifiable as information hot-spots in complex environments and fiducials can be easily deployed at a minimal cost [10,26]. Despite their advantages, the main limit of computer vision approaches is that localization is only available at sparse positions, *i.e.*, only when the phone observes a marker, and they cannot guarantee continuous tracking, thus requiring some form of active cooperation from the user.

Once user position is obtained, cultural contents related to the user location can be conveyed and displayed on the mobile guide. Contents are one of the main aspects for these guides, and thus they have to be properly designed and communicated to address the characteristics, interests and curiosity of the museum audiences [4–7]. In other words, each type of target audience requires a specific language and different insights to suit its features, a relevant aspect which has been underestimated in several approaches. The key to provide personalized access to the museum contents is exploiting context information to develop adaptive mobile guides. Examples are the Hippie project [27], where user's location as well as personal interests, knowledge and preferences are used to choose alternative paths inside the museum and to select the appropriate content to be presented, and the PEACH project [28], where a user model is automatically built while the user moves inside the museum and then used to adapt the displayed content and suggest additional elements of interest.

Adaptive museum guides can also take into account, as contextual parameter, the users' visit styles and derive recommendation algorithms from them. Visiting style models categorize visitors according to criteria such as route followed, dwell time per exhibit and number of stops inside the museum. On this basis, authors have proposed several classifications [29–31]. For instance, visitors' visiting patterns have been compared to different animal behaviors, identifying the *ant*, *fish*, *butterfly* and *grasshopper* styles [32]. Other scholars focused on the role of motivation in shaping visitors' paths [33,34], suggesting definitions such as *researchers*, *browsers*, *searchers* and *followers* ([35]), with the first two preferring to wander in the museum instead of following a suggested path. A different, and more subtle, approach to characterize the visiting styles consists in developing agent-based models of visitor behaviours, such as in [36]. In order to provide a satisfying and effortless experience, a mobile guide should also take into account that people's attitudes can change throughout the visit. According to the literature, cruising behavior frequently occurs when fatigue overcomes attention, especially for first time visitors, while people familiar with a specific venue might assume a more selective visiting style [37].

Another element to consider is that a museum is a social space [38]. Visiting a museum, living a cultural experience and learning while having fun are all activities that generally visitors want to share with friends and other companions. Some approaches addressed this issue. For instance, the iMuse Mobile Tour project [19] is a system that manages groups of visitors, identifying a group's leader that handle the main device and shares information and decisions with his/her visit companions. Other projects provide users with the possibility to actively take part in a social experience inside the museum by leaving feedback and comments/likes on the main social networks about the work of arts on display [21,39], sharing social games [16], leaving a public note for future visitors and exchanging synchronous messages with the current ones [15]. Hsu and Liao [20] even gave museum goers the possibility to have a personal microblog on their cultural experience, enhancing their level of active participation.

## 2. The Structure of MusA

In this section we describe the various features that are available in MusA to build interactive multimedia mobile guides for museum and cultural sites in general. As we stated in the Introduction, MusA is a general framework that focuses on two main issues curators and exhibit designers have to face: (i) supporting mobile users with information of interest related to the works of art on display or the surrounding environment and (ii) helping them to follow their visit path inside the venue.

Both these problems are addressed in our solution through the delivery of location based services, which rely on the accurate identification of the indoor location of the visitor by means of a vision-based positioning system. In the following subsections we describe the key features of MusA:

- indoor positioning;
- indoor navigation;
- the management of rich contents related to the cultural items of the exhibits;
- the design and management of compelling thematic paths;
- the integration of social networking features.

For each component and function we will point out its rationale and underline how the proposed solution is effective in the given context. MusA has been implemented in Android and its porting to iOS and other platforms is in progress. As a result of our work, we will also present the implementation of two different mobile applications based on MusA and discuss their evaluation through a user survey.

### 2.1. Our Vision-Based Approach to Indoor Positioning

Computing metric information from 2D images requires a fundamental step known as camera calibration. The aim of this process is to compute, for a given camera, both its extrinsic parameters (location and orientation of the camera in the world reference system) and intrinsic parameters (describing the relationship between pixel coordinates and camera coordinates). Camera calibration requires knowledge of the correspondences between a set of known 3D points and their projection on the image plane. These correspondences can be often obtained from known calibration objects, exploiting a set of 3D reference points [40,41], images of planar patterns under different perspectives [42,43] or sets of collinear points [44]. As an alternative, self-calibration techniques, relying only on the point correspondences between different images, can be used [45].

In a marker-based localization system, like the one used in our work, camera calibration aims at computing the camera pose from a single planar target. This process is often divided into two steps. First, the position and orientation of the sensing device, relative to the local reference system of a visual marker, are computed from the identification of the relevant marker features. Then, the absolute position of the device can be computed by knowing the feature positions in a reference frame.

The main problem to face in this case is that camera pose can be computed from at least four co-planar and not collinear points if the intrinsic camera parameters are known [46–48], while robust and full calibration from a single image still remains a challenge. Several algorithms have been proposed in the literature to solve this problem, exploiting either vanishing points [49,50], vanishing lines [51], conics [52,53] or the image of the Absolute Conic [42], usually making some assumptions on the intrinsic parameters (e.g., zero skew or known principal point) to reduce the complexity of the problem.

Given the possible application scenario of a MusA based mobile guide and the fact that it can be potentially run on any off-the-shelf mobile phone, we took into consideration the following points in the development of our pose estimation approach:

- the positioning algorithm should be as light as possible in order to be executed in real-time, even on devices offering limited computational power; hence, for these devices, approaches requiring the optimization of non-linear objective functions with complex and iterative algorithms (e.g., [46,54]) are not the best-suited;
- a lightweight algorithm, requiring a low amount of energy for its execution, helps reduce the device battery consumption, which is a vital parameter for any application aimed at supporting the visitor for a medium to long time span;
- any approach requiring one to know in advance the intrinsic camera parameters (e.g., [46,54,55]) is again unsuitable, since these parameters vary from camera to camera, thus requiring a database of camera parameter sets, periodically updated to consider new devices;
- in indoor positioning and navigation, the environment is usually represented by 2D maps and, thus, the localization data can be reasonably expressed with only three degrees of freedom [10]; another thing to consider is that, in most scenarios, a reliable indication of the user orientation inside a building is required to provide useful navigation information, while we can be satisfied with a less precise estimate of the device position, or even considered it as co-located with the detected marker.

With this in mind, we designed a novel visual marker, whose peculiar shape and geometric properties allowed to develop an approximate algorithm for the computation of the camera pose with the following characteristics:

- it does not require prior knowledge of the intrinsic parameters of the camera;
- it is not iterative, guaranteeing a fixed execution time;
- it is computationally light (*i.e.*, it can be executed in real-time on any mobile device), while ensuring, at the same time, a sufficient level of robustness and reliability for its expected use.

#### 2.1.1. The Structure of Our Marker

We designed our marker taking into consideration the following constraints: (i) providing the minimal amount of information required to solve the so called Perspective-n-Point (PnP) camera pose determination [56]; (ii) guaranteeing some geometric properties that help improving the accuracy and robustness of the camera pose estimation and; (iii) encode an ID into the marker shape.

**Figure 1.**
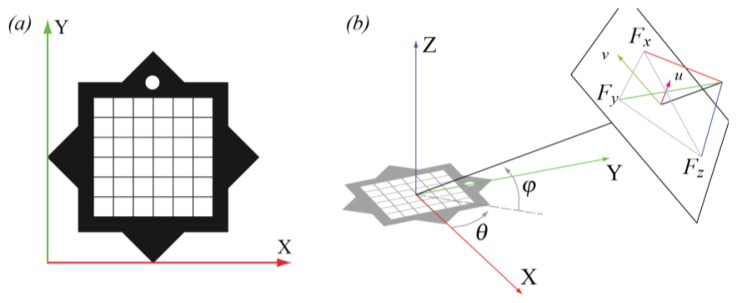
The structure of our marker (**a**) and the relative position of camera and marker (**b**).

Our star-shaped marker, sketched in Figure 1a, is planar and it is obtained by combining, on a white background, two black squares with the same side, the same center and mutually rotated by 45 degrees. The use of black and white colors for the marker guarantees an easier segmentation and feature extraction. The marker silhouette always contains, even under perspective distortion, a sequence of 16 alternate concave and convex corners. One of the triangles of the star includes a white circle, which allows one: (i) to define a local reference system aligned with the marker; and (ii) to univocally identify the projections of the marker corners on the image plane.

The inner part of the symbol contains the *data area*, divided into a regular grid of *N^2^* blocks each of which represents a data bit, where the extra information associated to the marker is stored.

#### 2.1.2. Marker Detection

The marker detection takes into account possible variations of illumination from frame to frame using an adaptive threshold to binarize the camera frame. In details, if a marker was detected in the previous frame, the threshold is the average of the highest and lowest intensities of its pixels, otherwise a random value is picked until a new marker is detected. After binarization, all the connected components bigger than a predefined area are treated as candidates and their contour is reduced to a set of vertices with the Douglas-Peucker algorithm [57]. A candidate is identified as a marker if its contour contains a sequence of exactly 16 alternate concave and convex corners and the characterizing white circle is found. This peculiar sequence of corners allows the use of larger thresholds in the Douglas-Puecker algorithm, making the corner detection more robust when compared with other convex markers (e.g., [10,54]).

The precision of the corner positions is finally improved considering that each of them lies at the intersection of two of the straight lines passing through the sides of the marker squares. These lines are computed, with the Total Least Square (TLS, [58]) method, as the lines best fitting the contour points between the four corners they traverse.

#### 2.1.3. Computing the Device Pose

Our libraries can compute the full 3D position and orientation of the camera relative to the marker, with an approach similar to the ones described in the literature. However, as stated in Section 2.1 we can reasonably express the visitor's indoor position and orientation with only three degrees of freedom. Thus, we designed a much lighter mobile positioning algorithm that takes advantage of the peculiar shape of our markers and of a straightforward simplification of the pose reconstruction problem.

In the following, we assume that the camera skew is null, the pixels are square and we approximate the location of the principal point with the image center. Let's choose the (*u*,*v*) image plane reference system having one of its axes parallel to the *xy* plane of the marker reference system (see Figure 1b). Then, the relative camera position can be written as a function of three parameters only: (i) the distance of the camera from the marker; (ii) the camera pitch (*i.e.*, the angle *φ* between the camera optical axis and the ground) and (iii) the camera yaw (*i.e.*, the angle *θ* between the projection of the optical axis onto the ground and the direction of the horizontal marker side). Then, according to the pinhole camera model, the position on the projection plane of the three principal vanishing points (*F_x_*, *F_y_*, *F_z_*) can be written as the following functions of the camera focal distance *f* and of the (*φ*,*θ*) camera orientation:

(1) $$\begin{array}{ccc} F_{x}=\left(\begin{array}{c} f\tan\theta /\cos\varphi \\ f\tan\varphi \end{array}\right), & F_{y}=\left(\begin{array}{c} -f\cot\theta /\cos\varphi \\ f\tan\varphi \end{array}\right), & F_{z}=\left(\begin{array}{c} 0\\ -f\cot\varphi \end{array}\right) \end{array}$$

From the geometry of our marker, it is easy to obtain the image coordinates of *F_x_* and *F_y_*. *F_z_* can be computed considering that the three principal vanishing points form a triangle, whose orthocenter is the principal point, approximated as the image center. Thus, the orientation (*φ*,*θ*) of the camera can be computed as:

(2) $$\begin{array}{cc} \theta =\tan^{-1}\sqrt{-\frac{F_{x,v}}{F_{z,v}},} & \varphi =\tan^{-1}\sqrt{-\frac{F_{x,u}}{F_{y,u}}} \end{array}$$

If we guarantee the marker is deployed having one of its sides parallel to the ground, the orientation of the camera in the world reference system can be computed from the output of the device accelerometer. Finally, the distance between the camera and the marker is estimated according to the size of the marker in the camera plane knowing the focal distance *f* in pixel units, which can be computed from the position of *F_x_* and *F_y_* as described in [50].

**Figure 2.**
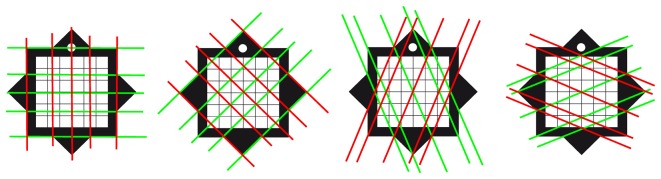
Different directions of parallel lines defined by the marker geometry.

The overall accuracy can be improved taking into account that the vanishing points of all the set of parallel directions in the marker plane lies on a line. Since at least eight of these directions can be obtained from our marker (Figure 2), the line best fitting their vanishing points can be used to compute a more accurate position of *F_x_* and *F_y_* and, consequently, to improve localization data.

This method is extremely light in terms of computational requirements. However, it suffers from the fact that for certain camera orientations, *i.e.*, when the camera plane is parallel to the marker plane, *F_x_* and *F_y_* go to infinity, and the algorithm cannot produce reliable results. To overcome the problem, when the camera is approaching this condition, its orientation is arbitrarily set to (π,*0*).

#### 2.1.4. System Deployment

Deploying the indoor navigation system in a new location requires different steps. First, we create a digital map of the environment with CAD software, exploiting already available CAD files or bitmapped maps of the venues. Then, we define the initial position and orientation of each marker and the possible routes in the building. Finally, marker and path data are stored into a database and the 2D vector maps are post-processed, to define their final graphic look and/or their 3D extension, as required by the application.

When markers are physically deployed on site, we refine their location information, revise the marker list in case some of them cannot be placed as defined in the first step, and update the database accordingly. Any further change to the database, due for instance to unavailability over time of certain segment paths or to marker relocations, is managed by updating the application local data when they are found to be obsolete.

### 2.2. Indoor Navigation and Path Communication

Routes are internally represented as a graph, where nodes are the turning points and edges are the path segments. The route between two points is computed with a shortest path algorithm. Besides user's location and target destination, the routing algorithm can take into account other contextual information. For instance, it considers the user preferences for stairs or elevator, the temporary availability of path segments (e.g., due to restricted opening hours of corridors, floors or venues), and the particular disabilities of the user, thus providing accessible routes for walking-impaired people.

**Figure 3.**
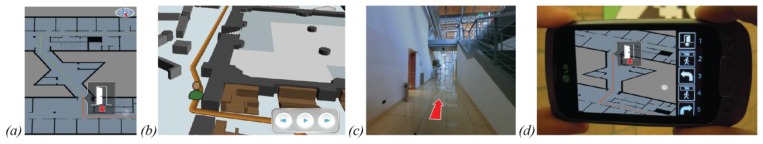
Communicating the route through: a 2D map (**a**); a perspective view of a 3D map (**b**); information in augmented reality (**c**); and a list of step by step instructions (**d**).

Once the route has been computed, one relevant question is which is the best way to describe this path. Communicating the route effectively enables users to easily find their way through spaces that sometimes they have never seen before. Thus, navigation information should be as clear and intuitive as possible, requiring a minimal cognitive load. In light of this, MusA offers the developers different presentation interfaces:

- A 2D (Figure 3a) or a 3D map (Figure 3b), showing users their current position and the path to follow. The displayed section of the map is adapted to the user's position and its graphical representation includes all the significant geometric and semantic elements in the surroundings. The map is oriented to show upward what is in front of the user. Since the precise user localization is available only at sparse points, *i.e.*, when the device camera observes a marker, an interactive graphical animation can be played to show the detailed turn-by-turn instructions to reach the destination.
- A visualization of navigation information in Augmented Reality [26] overlaying the device video with signs and arrows showing the current walking direction (Figure 3c). The AR view (as any other view) can be integrated with a list of graphics icons summarizing the step-by-step instructions to reach the destination ([26], Figure 3d).

As we experimented, the integration of some, possibly all, of these features improves the system usability and the effectiveness of navigation information, since map reading appears to be a major problem for most casual users.

### 2.3. Content Management

The cultural contents or any other kind of information related to the exhibit elements are prepared by the curators and the exhibit staff in a digital form. MusA offers the possibility to manage a large variety of different data and to organize them in many different ways. The system supports the following content types:

- text, composed by an HTML document and a CSS sheet defining its presentation semantic to allow better presentation flexibility and improved content accessibility (e.g., designing pages for dyslexic users);
- images, graphics, video and audio;
- high-quality 360° panoramic images, enhanced with embedded hotspots that, when selected, allow the linking of different types of digital media;
- 3D models, showing virtual reconstructions of objects, rooms or even buildings;
- Augmented Reality contents, registered with the camera view according to the positional data extracted from the markers.

Contents can be accessed in different ways. As a first option, entries in the content database can be linked to a marker ID and activated when the marker is recognized by the mobile guide. Alternately, contents can be directly reached from the application interface, e.g., through buttons, hyperlinks or hotspots.

In order to allow visitors to explore the information related to a specific item in as much detail as they like, contents can then be layered at different level of depths. Thus, for instance, curators can prepare an introduction with a limited amount of text followed by an in depth analysis, which relies on different combinations of accessory multimedia material (audio/video commentary, image galleries, references to other object inside or outside the collection, and so on). An XML file defines the list and organization of the contents related to a specific item and how they should be presented to users.

As for the storage policies, in order to deal with memory limitations, MusA supports in a transparent manner both the streaming of contents through a remote server and their storage in an internal database. The application designer can then choose the most suitable architecture according to different parameters, such as the memory requirements, the availability of reliable network connections and their bandwidth.

### 2.4. Exhibit Management

Different exhibit paths can be designed by curators and managed by the application. The paths include a number of selected stops, each corresponding to the exploration of a specific museum room or cultural object. Conversely, the works of art on display can be included in different thematic paths, which are played around specific topics identified by the curators. Thus, cultural objects and themes are combined in MusA in a matrix structure that allows to communicate the information of a specific object under a variety of perspectives, *i.e.*, according to the path theme or to the specific target group identified by the curators.

Different arrangements of the cultural objects include the possibility to offer free-choice approaches that allow visitors to organize contents according to their curiosity and interests and to make their own connections, thus building their own learning path.

Another relevant aspect to consider is that nowadays more people are visiting museums with the expectation to learn something, while having an entertaining experience. To this end, the thematic themes can also be played around narratives and interactive storytelling. Such narratives can be built on top of the elements supported by MusA, *i.e.*, combining video overviews or short stories, exploiting VR/AR contents to display objects otherwise not accessible or to show how a location looked at different periods in history [59–61] and comparing side by side paintings or sculptures that have common or contrasting traits [62]. Engaging characters can populate these stories and even interact with visitors at different levels [28,63,64]. The system can also support games and game-like experiences, which are becoming increasingly important to museums seeking to engage new audiences and to provide deeper engagement with existing ones, due to their ability to involve visitors in new and unexplored ways [65].

### 2.5. Social Media/Social Networking (Sharing and Profiling)

The networking capabilities of modern mobile devices enable to extend the museum experience to the universe of social networks. On the one hand, sharing comments and multimedia content with friends allows visitors to reinforce their experience and take active part into a process of participatory communication with the museum [66]. On the other hand, the act of sharing has a remarkable marketing potential, since it facilitates the spreading of the electronic word of mouth, it contributes to the reinforcement of the brand reputation of the museum and it may also play a positive role to reduce friends' hesitancy to visit a specific cultural venue [67,68].

To this end, MusA offers interfaces with the most popular social media platforms. Thus, users can exchange with their social companions commentary, notes, ideas and impressions on the collections.

Visitors can also be profiled. Information on the use of the application is stored within the guide, in strict anonymous form, for subsequent analysis about how users moved into the museums, which contents they accessed and how they used the guide. This analysis is a valuable support for exhibit curators, to fine-tune or modify their design according to the feedback received from visitors, and for the museum manager, to build up customer loyalty.

## 3. Analysis of a MusA Case Study

**Figure 4.**
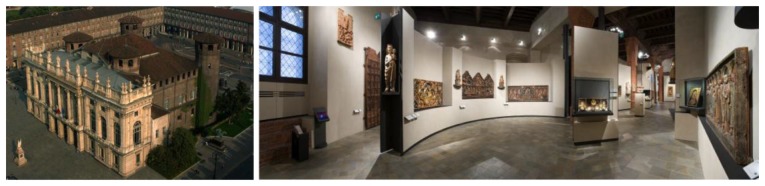
An image of Palazzo Madama (**left**) and of one of its venues (**right**).

The implementation and use of MusA in a museum context has been recently tested at Palazzo Madama—Museo Civico d'Arte Antica, a historic building and UNESCO World Heritage Site located in the city center of Turin, Italy (Figure 4). Palazzo Madama is especially renowned for its baroque style, but it also hosts the City Museum of Ancient Art (Museo Civico d'Arte Antica), which counts with an extensive collection of decorative and fine arts covering a span time of over eighteen centuries.

The museum's collections are organized in four categories over the four floors of the building: medieval stoneworks (moat level), Gothic and Renaissance masterpieces (ground floor), Baroque art (first floor) and decorative art objects, such as ceramics, textiles, glassware and ivories (last floor). This heterogeneity, combined with the extension of the venue over 4,000 m^2^, makes Palazzo Madama a particularly multilayered and complex cultural context: as a result, visitors have frequently reported difficulties orientating themselves inside the building. Additionally, previous studies have pointed out that only a minority of visitors actually follows the thematic and chronological paths designed by curators, showing critical issues with both physical and cognitive orientation [69].

Based on these observations, the authors deemed Palazzo Madama as being particularly suited for experimenting with the functionalities of MusA in a real and challenging scenario. To this end, two different applications were developed: “*Step by Step*”, a mobile museum guide addressed to an adult audience, and “*Intrigue at the museum*” a location-based game specifically designed for children. The two applications were deployed and thoroughly experimented in order to analyze the user experience and the visitors' appreciation of the applications.

### 3.1. Palazzo Madama Step by Step

*Step by step* is a mobile guide aimed at guiding visitors through the museum collection. Relying on MusA functionalities, three thematic paths were developed by exhibit designers:

- “*Great Treasures*”, devised for helping visitors with tight time constraints to discover the museum masterpieces;
- “*Discover the unusual*”, a selection of the museum's works of art that present uncommon and usually unnoticed characteristics;
- “*Decorative techniques*”, focused on the materials and the techniques mastered by artists over several centuries.

**Figure 5.**
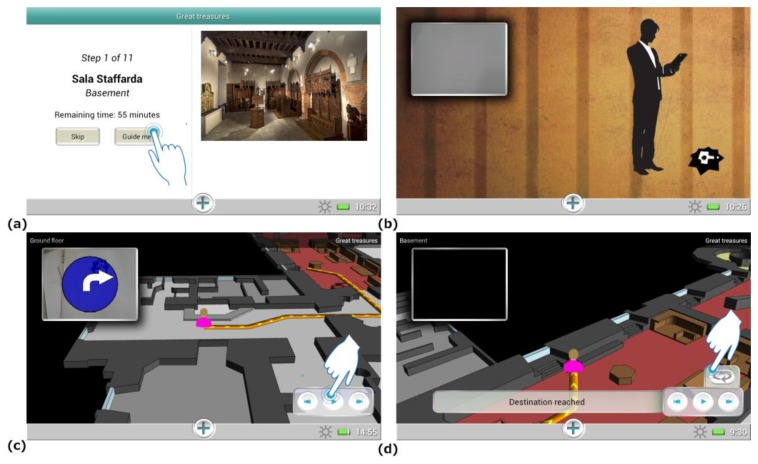
Screenshots of the application interface showing a step of the tour (**a**), the marker aiming menu (**b**), the 3D map and the path animation (**c**, **d**).

These paths were developed in order to cater to visitors' interests and visiting agendas. Additionally, considering that visitors have different visiting styles and may prefer not to be guided in the museum, users are also able to build their own personalized visiting path through the “*Wander and learn*” tool, which let them freely select the rooms and works of art to be explored. To provide maximal flexibility, users are allowed to switch the current visit theme at their will. The users can then be guided at any moment to their next destination (Figure 5a) activating the navigation function and pointing with the tablet at a MusA marker in their surroundings (Figure 5b). When the tag is recognized by the system, the navigation data are presented to the user on the tablet display. To make this information as clear and readily understandable as possible, route communication integrates, as suggested in Section 2.2, different elements: a 3D map, rotated according to the user orientation, and an arrow showing in AR on the camera image the current walking direction. The whole path is described on the map and the user can control an interactive animation showing the detailed instructions to reach the destination (Figure 5c,d).

When the desired destination is reached, the user can activate the related contents by pointing the tablet to the marker associated to that item or, alternatively, clicking a button in the user interface. The layered contents are then fetched from the DB and displayed on screen according to their structure.

**Figure 6.**
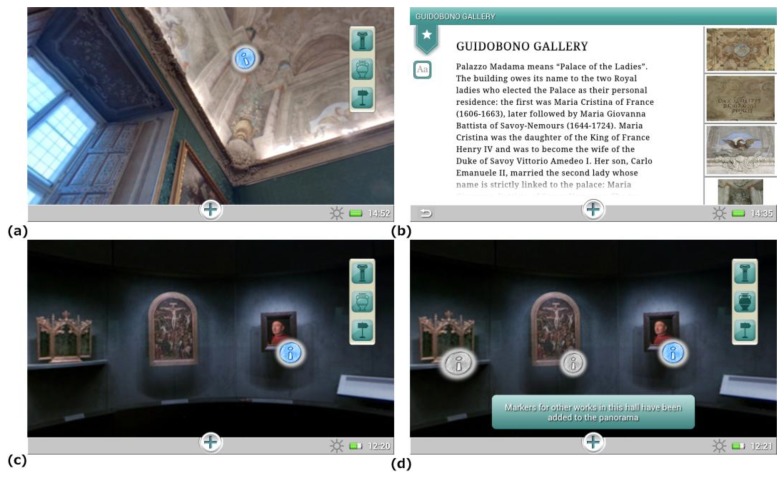
An interactive hotspot (**a**) and its related content (**b**); a panoramic image showing the path hotspots, in blue (**c**), as well as those included into alternative paths, in gray (**d**).

Visitors can access contents related to the environment and works of art on display also through hotspots embedded in interactive 360° panoramic images (Figure 6a,b). The rationale of integrating this feature into the guide is that the panoramic images allow a seamless shift between the image displayed and the surrounding environment, facilitating the interaction with the elements it contains [70]. Panoramic images also allow a reduction of the number of tags installed in the museum rooms. Furthermore, they offer visitors the possibility to display, and eventually access, other items present in the same room but not included in the thematic path chosen (Figure 6c,d), which can possibly foster visitors' interest into alternative paths in the museum collection.

**Figure 7.**
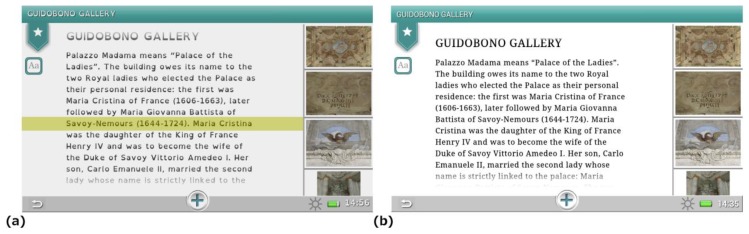
A screenshot of the interface for dyslexic users (**a**), which, compared to the version for non-dyslexic users (**b**), shows the usage of the OpenDyslexic font, a lowered contrast in the interface and the possibility to highlight the current reading line, helping users to focus on the text.

The whole application can be customized by users, selecting the display language, requesting accessible routes or modifying the interface setup, e.g., to display contents designed for dyslexic users (Figure 7).

### 3.2. Intrigue at the Museum—The Game

**Figure 8.**
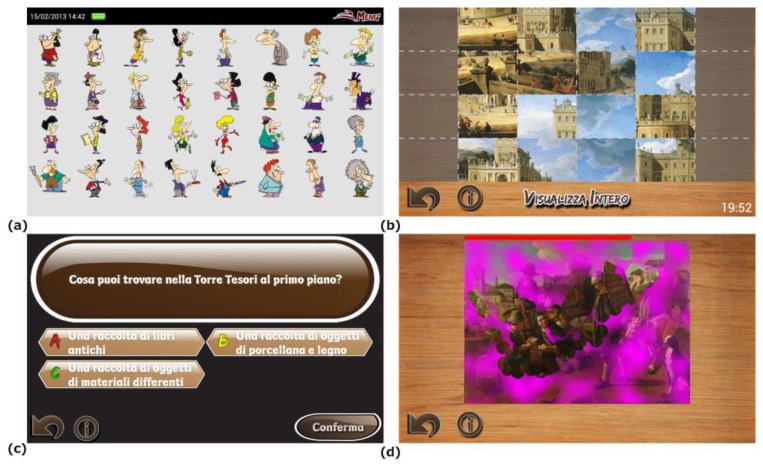
*Intrigue at the museum*: the list of suspects (**a**); examples of observation (**b**); reasoning (**c**) and arcade (**d**) mini-games.

*Intrigue at the museum* is a location-based mobile game targeting a young audience, *i.e.*, 7–13 years old children. Its goal is to identify a virtual thief in a set of suspects, with the help of different clues obtained by the player as he/she explores the environment solving puzzles and mini-games activated by the markers deployed in the building. These mini-games are related to the artworks or the museum venues (Figure 8). In order to progress in a mini-game, different pieces of cultural information received by players at the game start and a careful observation of the artworks and their surrounding environment are necessary, thus encouraging children to pay attention to details that could be easily unnoticed.

The mini-games were developed following the paradigm of Task Based Learning [71], and belong to one of these categories:

- *Observation tasks*, pushing players to carefully observe the masterpieces looking for the details needed to solve the game;
- *Reasoning tasks*, where initial clues, such as temporal information, factual details and so on, have to be absorbed by players in order to solve the riddles or quizzes;
- *Arcade tasks*, used to provide observation stimuli entertaining the players with animated graphics and high interaction.

This gamification approach has several objectives. First, it tries to make the museum visit more appealing, interesting and fun for younger visitors by letting them observe the museum collections from a different perspective. Second, it motivates players to explore the exhibit, since virtually all rooms have to be visited to collect an amount of clues sufficient for solving the main game. Third, it communicates educational contents and help visitors focus their attention on them.

## 4. Evaluation: Results and Discussion

Both *Step by Step* and *Intrigue at the Museum* were thoroughly tested after their deployment to analyze several elements of the user experience and to investigate the degree of appreciation of the applications and of their functionalities. Among the different methods used in current mobile usability research [72], we opted for a field study entailing the involvement and participation of actual visitors of the museum. Such approach allows to conduct the evaluation in a realistic environment, taking into account, among the factors usually reported as affecting the mobile experience [6,73,74], not only users' characteristics (*mobile user factor*), but also the environmental and social context of use (*mobile environment factor*). Overall, the analysis followed a mixed-method approach combining quantitative and qualitative research. Given the peculiar characteristics of the user categories targeted by the two applications, different protocols were applied in their evaluation process.

### 4.1. Evaluation of Step by Step

Volunteers were recruited at the museum ticket office, asking visitors to freely use the application and participate in the test. Each user was then provided with a 7 inch tablet to run *Step by Step*. In order to conduct the evaluation under realistic conditions, we preferred not to ask visitors to perform specific tasks, but rather to use the application according to their curiosity and interests. We gave some general information on the application, without explaining in details all its functionalities and let participants familiarize themselves with the use of the tablet through a sequence of introductory screens. Pre- and post-visit questionnaires were collected, followed by semi-structured interviews addressed to a subset of users. The panel was composed of 171 volunteers, 57% males and 43% females. The majority of participants were first-time visitors (75%). Visitors spanned different age groups: 36–50 (28%), 27–35 (26%), 18–26 (22%) and 51–65 (15%); participants younger than 18 years were only 5%, since they were invited to use *Intrigue at the museum*.

#### 4.1.1. Pre-Visit Questionnaires

The pre-visit questionnaire mainly aimed at investigating the *mobile user* factor by collecting socio-demographic data, visitors' expectations and information about their level of confidence with mobile devices. As for the approach to technology, most of respondents (70%) declared to own a tablet or a smartphone, 25% of them perceived her/his level of confidence with these devices as “excellent”, 40% as “good”, 23% as “average” and only 5% as “low”. Other results pointed out that most users decided to use the application attracted by new ways of exploring cultural heritage sites, desiring to get a better understanding of the museum exhibits. Interestingly, the possibility to support the navigation inside the museum, even if described as one of the application features, was scarcely mentioned among the expectations reported by volunteers.

#### 4.1.2. Post-Visit Questionnaires

**Figure 9.**
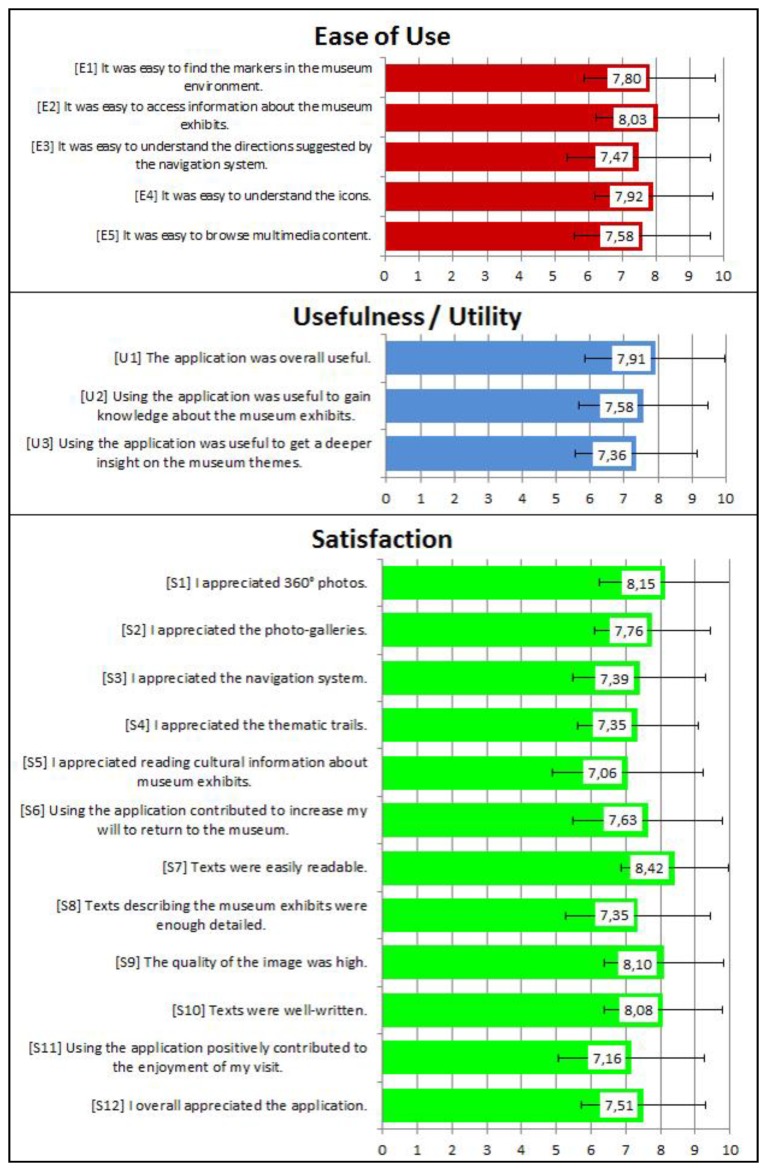
Post-visit questionnaires.

Post-visit questionnaires aimed at investigating the usability of the application, with specific regard to the *ease of use*, *usefulness* and *satisfaction* dimensions mentioned by the literature [73]. The questionnaire stimulated volunteers to express their level of agreement with a set of statements, using a 10-point Likert scale, or to make choices between options. Results are summarized in Figure 9 which report the most relevant questions related to the three dimensions of our usability framework, their average ratings and a vertical line indicating their standard deviation. Most of the answers were found to be consistent (standard deviation in the range [1.55, 2.17]).

The overall degree of satisfaction manifested by volunteers towards *Step by Step* was positive, with an average rating of 7.51 (S12). Among the functionalities enabling visitors to access and interpret information, 360° photos were the most appreciated feature (8.15, S1), most likely for their intuitive nature. Other multimedia features such as photo-galleries (S2) and texts (S5) were rated 7.76 and 7.06, respectively, with texts acknowledged as easily readable (8.42, S7).

The lower rating of textual contents might be explained taking into consideration that they were not specifically tailored for the mobile guide. Given the limited number of museum staff dedicated to the project, textual contents almost replicated those formerly elaborated for a printed guidebook and were not adapted to the device: for instance, they were definitely longer than the 600 characters that empirical studies underline as the limit to be fully read on a mobile device ([74]). Moreover, despite their verbosity and their perceived high quality (8.08, S10), some visitors did not find them detailed enough (7.35, S8). These results suggest that a better adaptation of the texts to the physical characteristics of the devices, the target audience and the context of use is sorely required.

The availability of different thematic trails (S4) was rated quite positively (7.35), suggesting that visitors generally appreciated the idea of potentially exploring the museum through a variety of perspectives, if so desired. Despite that, 76% of volunteers actually chose the free exploration tour. This suggests that the proposed themes were not fully matching visitors' motivations and interests and a better elaboration of trails is required to make this feature more appealing for the audience.

As for the *usefulness* dimension, users agreed that the application was useful overall (U1, 7.91), facilitating to a certain degree the acquisition of a better knowledge (U2, 7.58) and a deeper insight (U3, 7.36) on the history of the palace and the works of art on display.

Indoor positioning and navigation (S3) were overall appreciated, receiving an average score of 7.39. Additionally, the analysis of the *ease of use* dimension pointed out that participants found the navigation process quite easy to follow (E3, 7.47) and the markers easily recognizable inside the environment (E1, 7.80). While indoor positioning and navigation still represent for visitors unexpected features for a museum context, as shown by the pre-visit questionnaires, our findings suggest that people generally appreciate these services once exposed to them.

Further statistical analyses were performed to better understand to what extent the technological, user and environmental factors influenced some of the usability dimensions investigated. More specifically, the analysis aimed at answering the following research questions:

- *Is the volunteers' satisfaction correlated to their familiarity with mobile devices?* Cross-tabulations (Pearson χ^2^ test: 2.09, *p* = 0.352) pointed out that owning tablets or smartphones was not correlated to the degree of appreciation manifested by participants (S12, ratings above 7). This shows that, despite some limits highlighted by the evaluation, the interface of *Step by Step* was rather intuitive.
- *Is the acquisition of new knowledge correlated to the enjoyment of the visit?* (U2, S11) The correlation was found significant (r = 0.643; *p* < 0.001), suggesting that mobile guide contents may play a relevant role on visitors' enjoyment.
- *Is an enjoyable mobile experience correlated to visitors' will to return to the museum?* Our results (r = 0.733; *p* < 0.001) suggests that when the use of the mobile guide contributed to the enjoyment of the visit (S11), this also affected visitors' desire to return and visit the museum again (S6). These results seem particularly relevant both in terms of learning and marketing.

Finally, cross-tabulations highlighted a statistically significant relationship between the degree of satisfaction towards the use of the mobile guide (S12, ratings above 7) and the willingness to pay for the service (Pearson χ^2^ test: 152.43, *p* < 0.001). Interestingly, of the 75 participants who were not keen on paying for the use of the tablet, 63 answered negatively since they did not want to have any additional cost on the ticket (10 euros) and not because they perceived the mobile guide as poor or useless. These results seem particularly valuable in terms of marketing and should be taken into account when thinking about integrating mobile services into a museum context.

#### 4.1.3. Semi-Structured Interviews

A subset of 35 visitors was invited to participate to a semi-structured interview after the use of *Step by Step* to further investigate the usability dimensions, and to gain better insights into other aspects of the application use (Table 1).

Overall, the majority of respondents (31) actively used the guide throughout the visit. However, (question IA5) 25 out of 35 people regularly combined the use of the tablet with the traditional habit of relying on informative tools such as panels, labels and touch screens. Considering that only four people had previous experiences with mobile museum guides (question IA6), it can be inferred that the mobile guide was perceived as a novelty and that people had to adapt to this new visiting approach.

As for the contents, 26 people stated that using the mobile guide was useful to get a deeper knowledge of the palace and of the museum collections (IU2). However, only 29 users regularly read the texts about the works of art on display (IA1), and only 18 visitors regularly explored the photo-galleries or the other additional contents. Interviews made evident that the interface was not clearly pointing out the availability of such materials, thus highlighting an element to be improved.

Twenty-two visitors used (IA3) and found useful (IU1) the indoor navigation system. Other participants preferred to wander throughout the museum by themselves, asking for directions to museum assistants or looking at maps in paper format, if needed. Again, this behavior was mostly explained mentioning traditional visiting habits. Some users found difficult to understand their position on the digital map (IE1) or the directions to be taken (IE2). These problems are related, as previously commented, to the issue of map readability and route communication, which should still be improved.

Concerning the *satisfaction* dimension, 30 participants found the application pleasant to use (IS1) and would recommend it to a friend (IS2), describing themselves when using the app as “interested”, “focused” and “curious” (IS3). People who did not enjoy the use of *Step by Step* defined themselves as being “distracted”, since recalling the operating principles of the system was difficult and reading long texts was negatively affecting enjoyment.

Finally, answers about the *usefulness* of the guide for the management of the visiting time (IU3) highlighted two different patterns: people who followed the guided tours used the guide effectively as a time manager, while, on the other hand, people who selected the free tour generally pointed out that accessing rich multimedia content induced them to explore in more depth the collection, satisfying their interests and curiosity, thus staying in the museum longer than expected.

### 4.2. Evaluation of Intrigue at the Museum

Volunteers were recruited at the ticket office among the young visitors matching the target age group. Like for *Step-by Step*, we did not ask them to perform specific tasks and users familiarized themselves with the tablet through a set of pages introducing the story behind the game. *Intrigue at the museum* was then evaluated through unobtrusive observations of young players and post-visit questionnaires addressed to players' adult companions.

Unobtrusive observations involved 30 young visitors and were aimed at understanding how the use of the mobile game was integrated into children's exploration of the museum, if the application was perceived as an enjoyable tool and if it actually facilitated a mind-on encounter with the museum environment. We specially investigated the degree of engagement and enjoyment facilitated by the game since, besides being desirable outcomes in themselves, they are also precursors of learning [75].

Additionally, observations were useful to check if the gaming experience was fluid and if children actually followed the game mechanics (e.g., scanning tags, activating mini-games, progressively excluding characters from the list of the suspects and so on), thus conducting an implicit evaluation of the *ease of use* of the interface and of the application as a whole.

In order to code players' behaviors, verbal and non-verbal key-indicators of engagement were previously identified among the ones presented by the relevant literature [76,77] and then adapted to the context. Results pointed out that 83% of young players revealed one or more signals of engagement, the most frequent being hunting markers, walking faster, and pointing at markers while saying aloud sentences such as: “*Look! There's another one* [*i.e.*, *marker*] *over there!*”.

A positive and purposeful interaction with the museum environment, detected through proximity to the works of art, stops and extended gazes, was registered for 56% of the players, whereas in 20% of cases the young players were so concentrated on the game that they barely paid attention to the museum environment. This non-desirable behavior suggests that for some users the focus was indeed on the gameplay and on the technology. The remaining 24% of volunteers did not show detectable signals of focused interaction with the works of art, even though meaningful interactions cannot be excluded. Although at this stage of the research it was not possible to systematically check the acquisition of knowledge by participants, we often heard children reporting pieces of information that could be gained only through the gaming experience, which, thus, resulted in a learning facilitator, at least for a subset of players.

Concerning the *ease of use* dimension, most of the participants successfully used the application after familiarizing themselves with it at the beginning of the visit. Usage problems were reported only for two children who did not immediately understand how to operate the system and asked museum staff for help. We think this result highlights the intuitiveness of the user interface.

**Figure 10.**
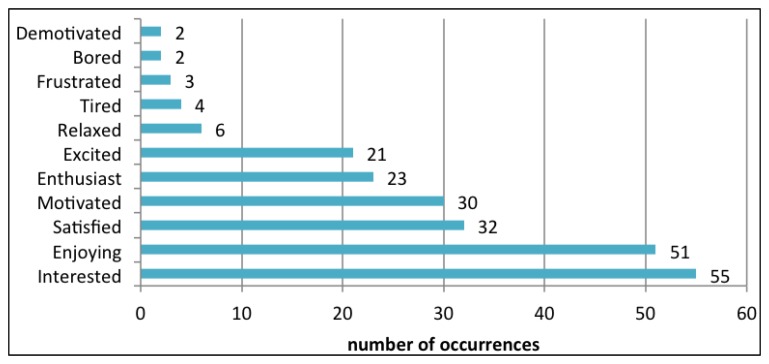
Adjectives describing players' feeling.

In order to investigate how adult companions perceived children's enjoyment, engagement and social interaction, 81 questionnaires were collected. The effectiveness of the game in facilitating an enjoyable visit is demonstrated by the adjectives used to describe the feelings manifested by children, which had almost exclusively a positive connotation (Figure 10). Answers also revealed that the application components more appreciated by players were, in order of relevance, solving the mini-games, hunting for the tags in the museum environment, finding the suspect and, finally, reading contents. This suggests that the application was successful in capitalizing on the physical exploration of the museum venue to foster attention and mind-on activities. The lower degree of appreciation manifested towards the contents was mainly related to the way they were communicated (*i.e.*, as texts), suggesting that better ways of integrating those contents into the game mechanics should be experimented.

## 5. Conclusions

In this paper we described MusA, an advanced multimedia system capable of supporting museum visitors with navigation information and cultural contents related to the artworks or places in their surroundings. MusA offers a set of location-based services, relying on a vision-based approach to detect users' indoor position and orientation. The combination of indoor navigation, path communication, contextual content delivery and exhibit management on a unique system, turned out to be a reliable and effective way to enhance visitors' cultural experience. Evaluation focusing on the usability of two mobile applications, based on the MusA framework and deployed in a museum, pointed out the overall appreciation of the services and the multimedia resources provided to enhance the visit. In particular, the high degree of satisfaction and engagement manifested by young visitors with regard to a mobile game addressed to children stressed the edutainment potential of gamification approaches based on location-based systems, suggesting how mobile applications could help museums turning into pervasive learning environments and enhancing visitors' cultural experiences.

As we experienced in both the described work and our on-going projects, MusA has shown itself to be a flexible and rich tool that allows the rapid prototyping and development of mobile guides for museum and cultural sites. Despite that, some critical points were identified during the evaluation of the test-cases.

While the cooperation required to activate a vision-based positioning system has not been perceived by our testers as a disturbing action, in some cases they reported problems to understand the navigation clues. This highlights a known issue, the proper communication of the path to follow, indicating that further research in this area is necessary. We are currently exploring alternative ways to present navigation hints, aimed at reducing the degree of abstract reasoning related to the navigation function. In particular, in our preliminary experiments, we found that the use of panoramic images to provide indications is a solution that appears to be promising, since users immediately match them with the surrounding world reducing the navigation cognitive load. As a result, we witnessed an improvement of the overall acceptance of indoor navigation. Further improvements are also required in the design of the application interface, since some users reported a cumbersome browsing of the different functionalities offered.

Finally, we experienced that the thematic paths should be carefully studied to meet the visitors' expectation and interests and that the contents, in particular the texts, should be fully adapted to the physical characteristics of the devices, the target audience and the context of use. These are not mere design problems, but require improvement of the communication between all the actors involved in the development process, expressly fostering the involvement of curators and cultural domain experts in the design cycle. To this end, we are actually developing specific tools, *i.e.*, an online graphical Content Management System, that lets them modify in an easy and intuitive way the path structures, the contents and their organization (layering and navigation), thus allowing them to play a more active role into the design process.

## Figures and Tables

**Table 1.** Outline of the semi-structured interviews.

**Ease of Use**

(IE1) *Was it easy to understand your position on the digital map?*
(IE2) *Was it easy to understand the direction to be taken to reach your next destination?*

**Usefulness**

(IU1) *Was using the navigation system useful to reach your desired destinations?*
(IU2) *Was using the mobile guide useful to get a deeper knowledge and understanding of the history of the palace and the works of art on display?*
(IU3) *Was using the mobile guide useful to better manage the time allocated to your visit?*

**Satisfaction**

(IS1) *Was it pleasant to use the mobile guide?*
(IS2) *Would you recommend the use of Step by Step to your friends?*
(IS3) *How did you feel when using the mobile guide?*

**Additional Questions**

(IA1) *Did you read the texts describing the works of art on display and the rooms of the palace?*
(IA2) *Did you use the photo-galleries*, *when available?*
(IA3) *Did you use the navigation system?*
(IA4) *To what extent have you used the mobile guide during your visit?*
(IA5) *Did you refer to informative tools such as panels*, *labels and touch screens during your visit?*
(IA6) *Had you already used a mobile guide in a museum context before today?*
